# Role of MicroRNA-145 in DNA Damage Signalling and Senescence in Vascular Smooth Muscle Cells of Type 2 Diabetic Patients

**DOI:** 10.3390/cells10040919

**Published:** 2021-04-16

**Authors:** Karen E. Hemmings, Kirsten Riches-Suman, Marc A. Bailey, David J. O’Regan, Neil A. Turner, Karen E. Porter

**Affiliations:** 1 Leeds Institute of Cardiovascular and Metabolic Medicine (LICAMM), University of Leeds, Leeds LS2 9JT, UK; k.e.hemmings@leeds.ac.uk (K.E.H.); k.riches@bradford.ac.uk (K.R.-S.); M.A.Bailey@leeds.ac.uk (M.A.B.); n.a.turner@leeds.ac.uk (N.A.T.); 2 Multidisciplinary Cardiovascular Research Centre (MCRC), University of Leeds, Leeds LS2 9JT, UK; david_o_regan@hotmail.com; 3 School of Chemistry and Biosciences, University of Bradford, Bradford BD7 1DP, UK; 4 Department of Cardiac Surgery, Yorkshire Heart Centre, Leeds General Infirmary, Leeds LS1 3EX, UK

**Keywords:** type 2 diabetes, saphenous vein, smooth muscle cells, DNA damage, senescence, microRNA, microRNA-145

## Abstract

Increased cardiovascular morbidity and mortality in individuals with type 2 diabetes (T2DM) is a significant clinical problem. Despite advancements in achieving good glycaemic control, this patient population remains susceptible to macrovascular complications. We previously discovered that vascular smooth muscle cells (SMC) cultured from T2DM patients exhibit persistent phenotypic aberrancies distinct from those of individuals without a diagnosis of T2DM. Notably, persistently elevated expression levels of microRNA-145 co-exist with characteristics consistent with aging, DNA damage and senescence. We hypothesised that increased expression of microRNA-145 plays a functional role in DNA damage signalling and subsequent cellular senescence specifically in SMC cultured from the vasculature of T2DM patients. In this study, markers of DNA damage and senescence were unambiguously and permanently elevated in native T2DM versus non-diabetic (ND)-SMC. Exposure of ND cells to the DNA-damaging agent etoposide inflicted a senescent phenotype, increased expression of apical kinases of the DNA damage pathway and elevated expression levels of microRNA-145. Overexpression of microRNA-145 in ND-SMC revealed evidence of functional links between them; notably increased secretion of senescence-associated cytokines and chronic activation of stress-activated intracellular signalling pathways, particularly the mitogen-activated protein kinase, p38α. Exposure to conditioned media from microRNA-145 overexpressing cells resulted in chronic p38α signalling in naïve cells, evidencing a paracrine induction and reinforcement of cell senescence. We conclude that targeting of microRNA-145 may provide a route to novel interventions to eliminate DNA-damaged and senescent cells in the vasculature and to this end further detailed studies are warranted.

## 1. Introduction

The mechanisms underlying increased risk of coronary heart disease (CHD) and its attendant complications in Type 2 diabetes (T2DM) are poorly understood. T2DM reportedly confers up to 15 years of aging [1] whilst chronological aging, characterised by loss of replication-competent cells and DNA damage, is a major risk factor for CHD. It is well known that overwhelming DNA damage triggers a permanent DNA damage response (DDR) and consequent cellular senescence, a permanent arrest of cell proliferation that is a fundamental mechanism of aging that may also play a role in some of the cardiovascular complications of T2DM [2].

Patients with significant CHD frequently require arterial reconstruction in the form of coronary artery bypass grafting (CABG), which in individuals with diabetes is most frequently performed using autologous saphenous vein (SV) grafts [3,4]. Phenotypic modulation of the venous smooth muscle cells (SMC) is an important mechanism that enables vein graft adaptation to an arterial environment. Indeed, effective integration to an environment of increased flow and pressure early after implantation is a key determinant of the long-term patency of SV grafts [5]. SMC are not terminally differentiated and it is their ability to retain plasticity, i.e., to modulate their phenotype in response to environmental cues, which is key to their role in maintaining vascular homeostasis. Our group previously discovered a distinct and persistent aberrant phenotype, specifically in SV-SMC cultured from T2DM patients compared with those cultured from age-matched non-diabetic (ND) patients that persisted throughout passaging [6,7,8]. The enlarged, flattened morphology and impaired proliferation of T2DM-SMC bore resemblance to those of aged, DNA damaged and senescent cells. However, the deleterious effects of DNA damage, senescence and subsequent acquisition of a senescence-associated secretory phenotype (SASP) in SV-SMC, specifically in the setting of human T2DM, have not been explored.

More recently we pursued the notion that persistence of a T2DM SV-SMC phenotype in long-term culture was suggestive of “metabolic memory” underpinned by an epigenetic mechanism. Indeed, we demonstrated that this phenotype was driven by elevated expression levels of a specific SMC-enriched microRNA, miRNA-145 [8]. Some recent studies have shown that miRNAs, short non-coding RNAs that are negative regulators of gene expression, in addition to regulating a host of cellular functions, also play crucial roles in cellular responses to DNA damage (reviewed in [9]). Dysregulated miRNAs are also believed to contribute to accelerated aging syndromes [10] and some vascular pathologies [11,12,13], yet little is known about any relationship between miRNAs, diabetes and vascular SMC dysfunction. Lack of consistency among cell types has suggested that dysregulation of miRNAs after DNA damage is cell-type specific [14]. The persistent aberrant phenotype and accompanying elevated levels of miRNA-145 that we regularly observe in T2DM SV-SMC [8] led us to hypothesise that a miRNA-145-mediated mechanism underlies defective DNA damage/repair pathways and subsequent cellular senescence, specifically in this cell-type.

## 2. Materials and Methods

### 2.1. Smooth Muscle Cell (SMC) Culture

SMC were cultured as previously described [15] from explants of SV obtained from non-diabetic patients (ND-SMC), or patients diagnosed with type 2 diabetes (T2DM-SMC) receiving oral therapy either alone or with the addition of insulin. All patients were undergoing elective CABG surgery at the Leeds General Infirmary or Nuffield Health Leeds hospital and samples were collected with local ethical committee approval (CA01/040) and informed, written patient consent. The study conformed to the principles outlined in the Declaration of Helsinki. SV specimens from a total of 36 patients were used: 19 ND (90% male, mean age 65.7 ± 2.2 years) and 17 T2DM (88% male, mean age 67.7 ± 2.7 years). T2DM patients were all receiving oral therapy, alone or in combination with insulin. SMC were cultured in DMEM containing 10% bovine FCS, 25 mmol/L glucose, 1% penicillin, streptomycin, fungizone and 2 mmol/L glutamine (full growth medium; FGM) at 37 °C in 5% CO_2_ in air. All experiments were performed on cells between passages 3 to 6.

### 2.2. Senescence Associated β-Galactosidase Staining

SMC were seeded at a density of 75000/well in 6 well plates and cultured in FGM for 48 h prior to fixation and staining using a commercial β-galactosidase assay (Cell Signaling Technologies #9860, New England Biolabs, Ipswich, MA, USA), as described previously [16]. Senescent cells were identified by formation of a blue precipitate at pH 6 and senescence scores determined by counting the proportion of senescent cells contained within 10 low power (×40) microscopic fields of view.

### 2.3. Quantitative Real-Time RT-PCR

Cellular RNA was extracted and cDNA prepared as previously described [17]. Real-time RT-PCR was performed in triplicate using an Applied Biosystems 7500 Real-time PCR system and Taqman Gene Expression Assays (Thermo-Fisher Scientific, Waltham, MA, USA). Data are presented as percentage of endogenous control (*GAPDH*) expression using the formula 2^−∆CT^ × 100.

### 2.4. Apoptosis Assay

SMC were plated in FGM at a density of 3000/well in 96 well plates and incubated overnight. Apoptosis assays were performed as previously described [16]. Briefly, cells were treated with 5 μmol/L NucView™ 488 caspase-3 substrate (Biotium). Images were obtained in phase contrast and fluorescence mode using a ×10 objective and an IncuCyte FLR time-lapse fluorescence microscope (Essen Bioscience, Newark, CI, USA). After 24 h, cells were stained with 1 μmol/L Vybrant DyeCycle Green^®^ (Molecular Probes, Invitrogen, Eugene, OR, USA) and an apoptosis index calculated using an inbuilt algorithm.

### 2.5. Immunocytochemistry

SMC cultured on glass coverslips were fixed in 4% PFA prior to immunostaining with phospho-histone H2AX (γH2AX) antibody (Cell Signaling Technologies #2577, New England Biolabs, Ipswich, MA, USA) as described previously [18]. Coverslips were mounted using Prolong Gold antifade reagent containing DAPI (Thermo-Fisher Scientific, Waltham, MA, USA) and imaged on a Zeiss 700 confocal microscope. Nuclear morphology was examined in DAPI-stained cells at × 400 magnification and the proportion of cell nuclei displaying DNA damage (blebbing or apoptotic nuclei) was quantified as the number of γH2AX-positive nuclei (pink) relative to the total number of regular, ovoid nuclei (blue).

### 2.6. Enzyme Llinked Immunosorbent Assays (ELISA)

Cell culture supernatants were collected from SMC, snap frozen and stored at −80 °C. Secretion of IL-6, IL-8 and MCP-1 was determined by ELISA (Quantikine, R&D Systems).

### 2.7. Cell Proliferation

Cells were seeded in 24-well plates at a density of 10,000 cells/well and triplicate counts performed over a 7-day period using a haemocytometer and trypan blue exclusion, as previously [6].

### 2.8. Immunoblotting

Whole cell homogenates were prepared and immunoblotted as described previously [17] using antibodies to p-p38 (#9215), total p38 (#9228), p-ERK (#9106), total ERK (#4695), p-Akt (#4691), total Akt (#2920), IκB (#9242), p21 (#2947), p-p53 (ser15) (#9284) (all Cell Signaling Technologies), with α-tubulin (ab8226; Abcam) as a loading control.

### 2.9. Quantification of miRNA-145 Levels

Total RNA samples were analysed by preparing specific RT reactions for *U6* and *miRNA-145* using Taqman MicroRNA Assays (Applied Biosystems). Real-time RT-PCR was performed in triplicate and data presented as percentage endogenous control (*U6*) expression using the formula 2^−∆CT^ × 100.

### 2.10. Inhibition of DDR Apical Kinases

Cells were seeded in FGM at 10,000 cells/well in 24 well plates, cultured overnight and then serum-deprived for 72 h prior to re-addition of FGM plus inhibitors of ATM (KU55933, 10 μM), ATR (AZ20, 10 μM), DNA-PK (NU7026, 3 μM) (all Generon) or DMSO vehicle.

### 2.11. siRNA Knockdown

SMC were transfected with siRNA to ATM, ATR, DNA-PK or scrambled control (20 nM, ONTARGETplus siRNA, Dharmacon) using Lipofectamine 2000 reagent (Thermo Fisher Scientific), according to the manufacturer’s instructions.

### 2.12. miRNA-145 Overexpression

Overexpression of miRNA-145 was achieved by transfecting SMC with 15 nM premiRNA-145 or negative control (premiRNA–ve) as described previously [8].

### 2.13. Statistical Analysis

Statistical analysis was performed using GraphPad Prism 7 software. Data are presented as mean ± SEM with *n* representing the number of experiments performed on cells from different patients. Data were tested for normality prior to log transformation and analysis by paired or unpaired t-test, or one way ANOVA with Dunnett’s post hoc test, as appropriate. *p* < 0.05 was considered significant.

All data are contained within the article and supporting information. Data are available upon request.

## 3. Results

### 3.1. Inherent Characteristics of Senescence and DNA Damage in Native SV-SMC

**Figure 1:**
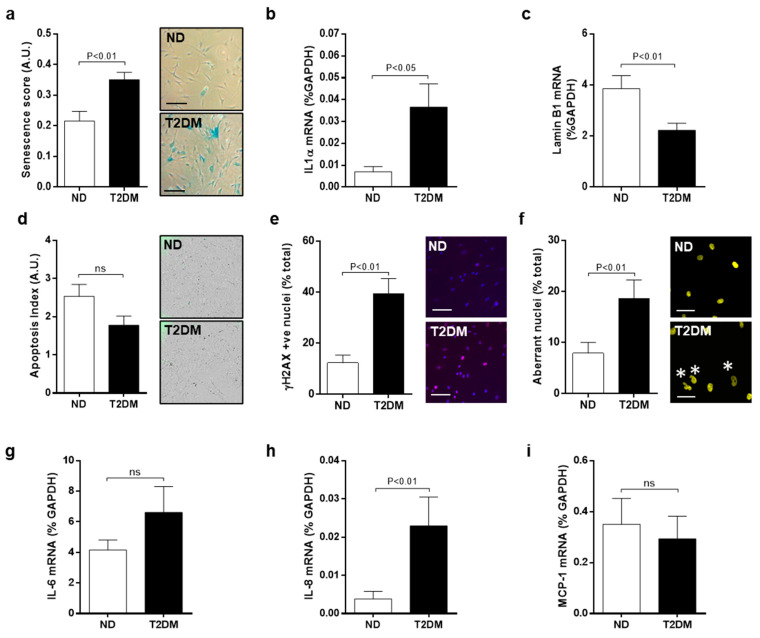
T2DM-SMC exhibit senescent features. (**a**) SMC were cultured in full growth medium for 48 h, fixed and stained for senescence-associated β-galactosidase (blue precipitate, scale bar = 200 μm, *n* = 7). (**b**) RNA was extracted from cells cultured for 24 h and expression of senescence-associated markers *IL-1α* and (**c**) *LMNB1* measured by RT-PCR (*n* = 8). (**d**) SMC were cultured in full growth medium for 48 h, fixed and incubated with NucView 488 caspase 3 substrate (to indicate apoptotic cells, *n* = 4), (**e**) stained for early DNA damage marker γH2AX (pink foci, scale bar = 100 μm, *n* = 8), and (**f**) DAPI to visualise aberrant nuclei (denoted by asterisk, scale bar = 50 μm, *n* = 8). Basal expression of senescence-associated inflammatory genes (**g**) *IL-6*, (**h**) *IL-8* and (**i**) *MCP-1* was measured using RT-PCR (all *n* = 8).

We previously reported impaired proliferation and an enlarged flattened morphology characteristic of senescence, in SV-SMC cultured from T2DM patients [6] which persists in culture and throughout passaging [8]. Given that there is no single and definitive hallmark of senescence [19], we examined a number of additional endpoints. T2DM-SMC exhibited significantly increased senescence-associated (SA) β-gal staining (Figure 1A), increased expression of *IL-1α* mRNA (Figure 1B), and reduced expression of nuclear lamin B1 mRNA (*LMNB1*) [20] (Figure 1C), relative to ND-SMC. No differences were observed in apoptosis (caspase-3 fluorescence assay) (Figure 1D). In addition, a significantly increased frequency of γH2AX positive nuclei, symbolic of double-stranded DNA breaks (Figure 1E) and aberrant nuclear morphology (Figure 1F) in the T2DM cells was indicative of augmented DNA damage. Expression of *IL-8* mRNA, an inflammatory mediator associated with SASP, was elevated; however additional SASP mediators *IL-6* and *MCP-1* were unchanged at the mRNA level (Figure 1G–I). Secretion of IL-6, IL-8 and MCP-1 from native ND and T2DM-SMC was not significantly different but considerable variability between patients was observed, independent of diabetic status (Supplementary Figure S1). These data provide evidence that SV-SMC cultured from patients with T2DM exhibit inherently higher levels of senescence and DNA damage than those of ND patients.

### 3.2. DNA Damage and DDR Pathway Activation in SV-SMC

**Figure 2:**
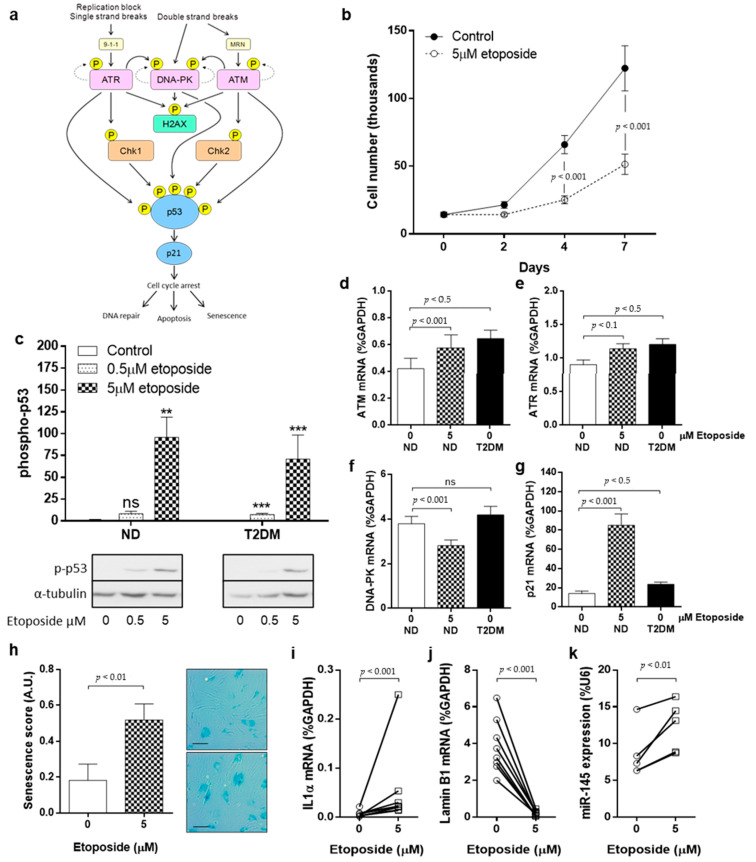
DNA damage drives a senescent SMC phenotype. (**a**) The DNA damage signalling pathway leading to phosphorylation of p53, and subsequent downstream effects. (**b**) SMC were treated with DNA-damaging agent etoposide (5 μM) or vehicle control (DMSO) for up to 7 days and proliferation quantified by cell counting (*n* = 7). (**c**) The impact of etoposide on p53 phosphorylation was monitored using Western blotting after 24 h (*n* = 4). (**d**) The effect of DNA damage on apical kinase expression was explored in ND-SMC that were treated with 5 μM etoposide for 24 h. The expression of *ATM*, (**e**) *ATR*, (**f**) *DNA-PK* and (**g**) *p21* was quantified using RT-PCR. The expression of these kinases was also measured in untreated T2DM-SMC to determine whether DNA damage per se could mimic a T2DM-SMC phenotype (all *n* = 8). (**h**) Cells were treated with etoposide for 24 h and then placed into FGM for 72 h. Cells were stained with SA-β-galactosidase (scale bar = 100 μm). (**i**) The influence of etoposide on expression of *IL-1α*, (**j**) *LMNB1* (both *n* = 8) and (**k**) *miRNA-145* expression (*n* = 6–8) was quantified using RT-PCR.

We next examined whether DNA damage alone could drive the acquisition of a T2DM-SMC phenotype in ND-SMC. Exposure to environmental stresses induces activation of the DDR signalling pathway, a cascade of kinase activations which culminate in cell cycle arrest to enable DNA repair. Phosphorylation of apical kinases ATM (ataxia-telangiectasia mutated), ATR (ATM- and Rad3-Related) and/or DNA-PK (DNA-dependent protein kinase catalytic subunit), depending on the type of DNA damage, activates multiple downstream events that lead to phosphorylation of H2AX (γH2AX) and the tumour suppressor gene p53, and expression of the cell cycle inhibitor p21 that confers growth arrest (Figure 2A).

DNA damage was induced in ND-SMC using the chemotherapeutic agent etoposide (Sigma-Aldrich), which led to a marked reduction in cell proliferation (Figure 2B) and increased phosphorylation of p53 (Figure 2C). Whilst *ATM* and *ATR* mRNA were expressed basally at higher levels in T2DM-SMC than in ND cells, exposure of ND cells to etoposide induced increases in both *ATM* and *ATR* mRNA, comparable to the levels of expression in native T2DM-SMC (Figure 2D,E). Conversely, *DNA-PK* did not differ between ND and T2DM-SMC and etoposide treatment caused a reduction in *DNA-PK* expression (Figure 2F). Basal gene expression of *p21* was inherently higher in T2DM cells and etoposide treatment of ND cells led to a marked increase in *p21* mRNA levels (Figure 2G). Markers of senescence, namely increased SA β-gal staining (Figure 2H), increased *IL-1α* mRNA expression (Figure 2I) and reduced *LMNB1* mRNA expression (Figure 2J), were observed in ND-SMC in response to etoposide, consistent with features detected in native T2DM-SMC (Figure 1A–C). Finally, ND-SMC treated with etoposide exhibited a 1.5-fold increase in *miRNA-145* expression (Figure 2K), consistent with differences we previously reported between native ND- and T2DM-SMC in a cohort of 130 patients [8]. *miRNA-145* expression was also induced by oxidative stress, a more physiologically relevant stimulus (Supplementary Figure S2).

### 3.3. Pharmacological Inhibition of DDR Kinases

**Figure 3:**
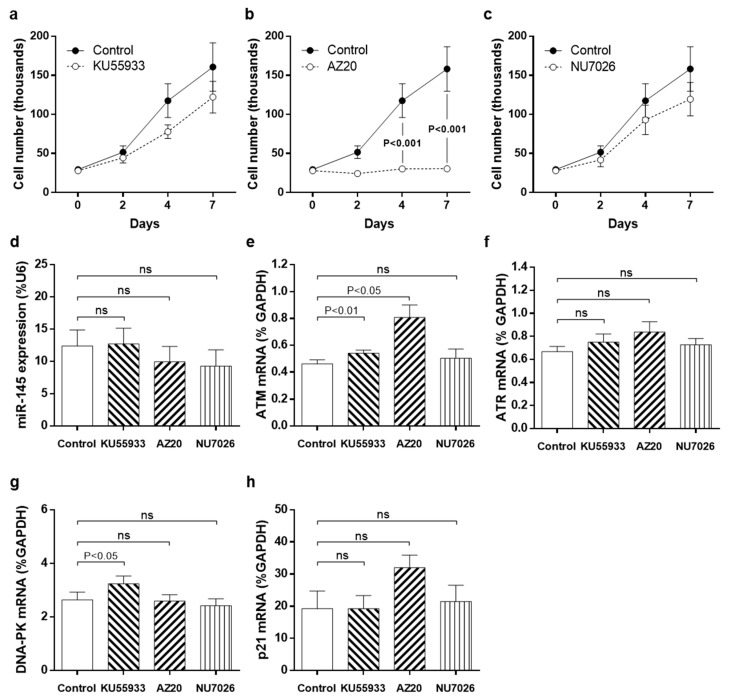
Pharmacological inhibition of apical kinases of the DNA damage response pathway modulates proliferation and gene expression. (**a**) SMC were treated with ATM inhibitor (KU55933; 10 μM), (**b**) ATR inhibitor (AZ20; 10 μM) or (**c**) DNA-PK inhibitor (NU7026; 3 μM) for up to 7 days and cell counts performed on days 0, 2, 4 and 7 (*n* = 4). After 72 h, RNA was extracted and the effect of all inhibitors on expression of (**d**) *miRNA-145*, (**e**) *ATM*, (**f**) *ATR*, (**g**) *DNA-PK* and (**h**) *p21* was measured using RT-PCR (all *n* = 6). ns = non-significant.

To examine whether elevated DDR expression was responsible for the T2DM-SMC phenotype, we employed a pharmacological approach in ND-SMC to investigate the effect of apical kinase inhibition on SMC proliferation. Whilst inhibition of ATM (KU55933) or DNA-PK (NU7026) did not modulate proliferation (Figure 3A–C), ATR inhibition (AZ20) completely abolished any increase in cell number over a 7-day period (Figure 3B). miRNA-145 expression was unaffected by any of the inhibitors (Figure 3D). Surprisingly, inhibition of ATM or ATR increased *ATM* mRNA expression (Figure 3E). None of the three inhibitors affected *ATR* mRNA expression levels (Figure 3F) and inhibition of ATM significantly increased *DNA-PK* gene expression by 23% (Figure 3G). Inhibition of ATR suggested a possible trend to increased *p21* mRNA expression (Figure 3H).

### 3.4. siRNA Knockdown of DDR Kinases

**Figure 4:**
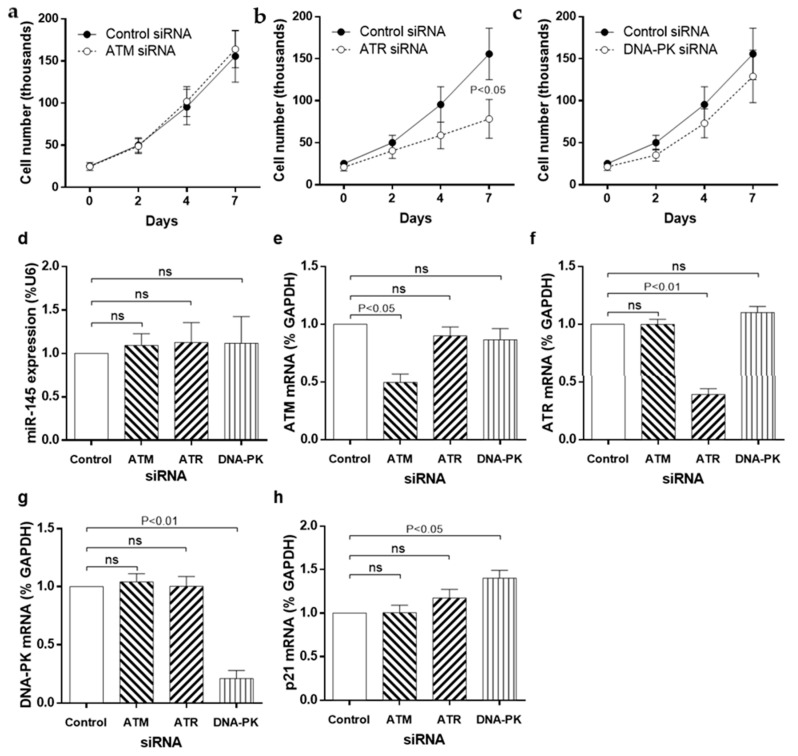
siRNA knockdown of apical kinases of the DNA damage response pathway modulates proliferation and gene expression. (**a**) SMC were transfected with siRNA targeted to ATM, (**b**) ATR or (**c**) DNA-PK for 6 h and then placed into FGM for up to 7 days. Cell counts were performed on days 0, 2, 4 and 7 (*n* = 4). RNA was extracted at 72 h and the expression of (**d**) *miRNA-145*, (**e**) *ATM*, (**f**) *ATR*, (**g**) *DNA-PK* and (**h**) *p21* measured using RT-PCR (*n* = 5).

To confirm these pharmacological data, we employed a gene-silencing approach to individually knock down ATM, ATR and DNA-PK. siRNA inhibition caused selective and specific reduction of each target mRNA within 24 h and was maintained for at least 96 h (Supplementary Figure S3). Anti-proliferative effects were comparable with pharmacological inhibition, specifically that ATM and DNA-PK knockdown did not modulate SMC proliferation (Figure 4A–C) whilst ATR knockdown led to a significant reduction in cell number (Figure 4B). As with pharmacological inhibition, *miRNA-145* expression was unaffected by ATM, ATR or DNA-PK knockdown (Figure 4D). Notably, the gene silencing approach enabled selective inhibition of each kinase, without off-target effects on the others (Figure 4E–G). In agreement with the pharmacological inhibition data, the effect of ATR knockdown on *p21* expression was again negligible, although increased *p21* mRNA expression was observed as a result of DNA-PK silencing (Figure 4H).

### 3.5. miRNA-145 Overexpression Modulates DNA Damage Signalling and Senescence

**Figure 5:**
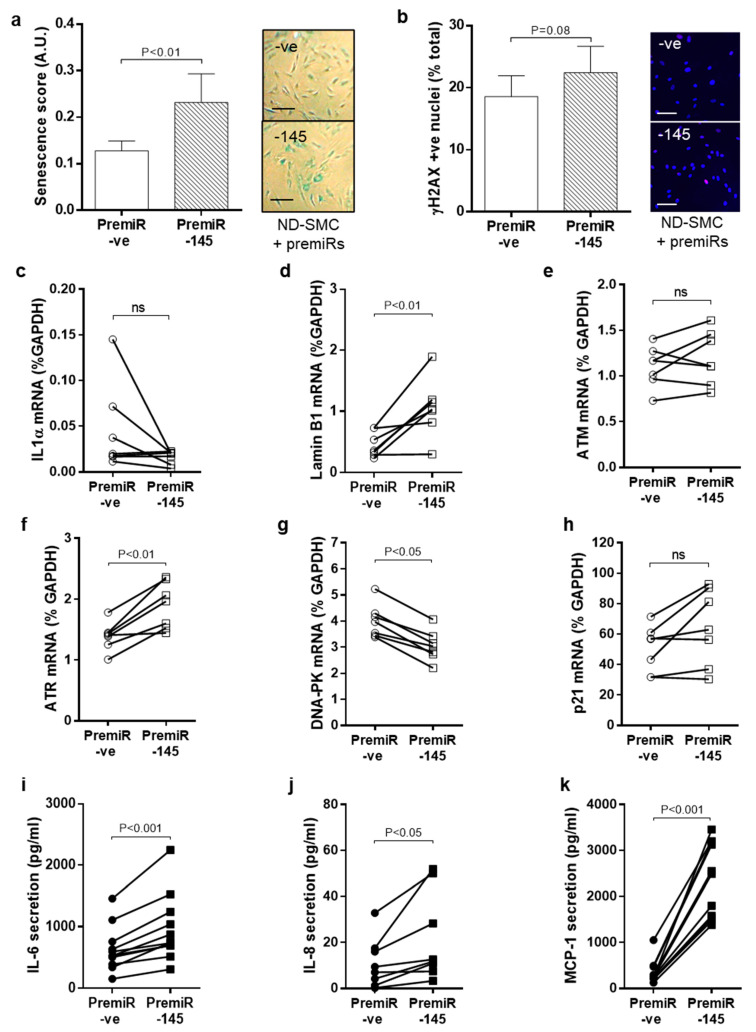
Effect of miRNA-145 overexpression on SMC senescence, DDR signalling and SASP. ND-SMC were transfected with premiRNA-145 or premiRNA -ve. (**a**) After 4 days in full growth media, cells were fixed and stained for SA-β-galactosidase (scale bar = 200 μM, *n* = 6). (**b**) *IL-1α* and (**c**) *LMNB1* expression were measured by RT-PCR after 72 h (*n* = 7). (**d**) Cells were treated as (**a**) and stained for γH2AX (pink foci, scale bar = 100 μM, *n* = 6). (**e**) Expression levels of *ATM*, (**f**) *ATR*, (**g**) *DNA-PK* and (**h**) *p21* were quantified by RT-PCR 72 h after miRNA-145 transfection (*n* = 7). (**i**) Protein secretion of IL-6, (**j**) IL-8 and (**k**) MCP-1 was measured by ELISA after 96 h (*n* = 11).

To determine whether miRNA-145 was upstream or downstream of DDR, ND-SMC were transfected with premiRNA –ve (negative control) or premiRNA-145 (overexpression) and parameters pertinent to the DNA damage pathway were evaluated. PremiR-145 transfection led to a marked overexpression as expected (Supplementary Figure S4) which remained elevated for ≥7 days. Overexpression of miRNA-145 led to increased SA β-gal 96 h post transfection (Figure 5A), although differences in γH2AX foci were less evident (*p* = 0.08, Figure 5B). In contrast to increased *IL-1α* and reduced *LMNB1* gene expression observed in native T2DM cells, the opposite was observed in miRNA-145 overexpressing cells; reduced *IL-1α* mRNA and increased *LMNB1* mRNA (Figure 5C,D). With respect to apical kinases, whilst no consistent differences in *ATM* gene expression were observed (Figure 5E), increased mRNA expression of *ATR* (Figure 5F) and decreased mRNA expression of *DNA-PK* (Figure 5G) were clear. Changes in *p21* expression following miRNA-145 transfection were inconsistent (Figure 5H). Importantly, secreted levels of IL-6 (52% increase; Figure 5I), IL-8 (99% increase; Figure 5J) and MCP-1 (674% increase; Figure 5K) 96 h after miRNA-145 transfection were unequivocally higher.

### 3.6. Chronic Signalling Pathway Activation in miRNA-145-Overexpressing SMC

**Figure 6:**
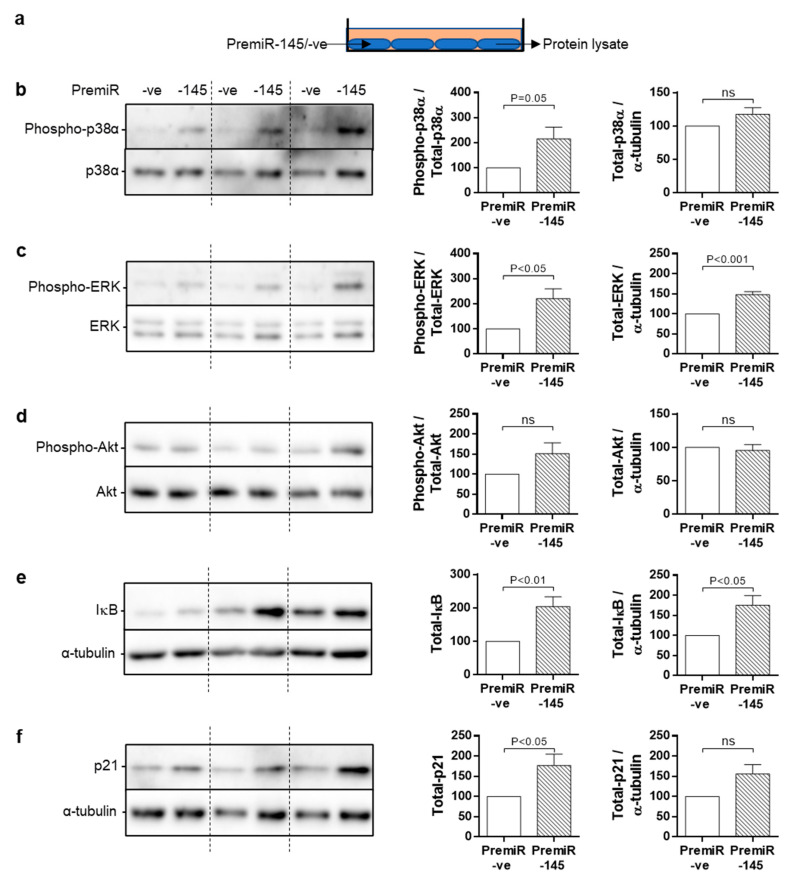
miRNA-145 overexpression induces chronic activation of intracellular signalling. (**a**) Cells were transfected with premiRNA-145 or premiRNA -ve for 6 h, placed into 0.4% FCS for 96 h and protein lysates prepared. These were immunoblotted for (**b**) p38, (**c**) ERK, (**d**) Akt (all expression and phosphorylation), (**e**) IκB, (**f**) p21 expression. Phosphorylation of proteins was measured against expression of the same protein, whereas changes in expression only were measured against α-tubulin as a loading control (*n* = 6–9).

Persistently elevated levels of miRNA-145 in T2DM-SMC may drive chronic stress signalling to reduce proliferation. To this end we explored activation of four intracellular signalling pathways, p38MAPK, ERK, PI3K/Akt and NF- κB. Cell lysates were prepared from ND-SMC transfected 96 h earlier with premiRNA-145 or premiRNA –ve (Figure 6A). Phosphorylation of p38α was consistently elevated in *miRNA-145* overexpressing cells, independent of altered p38 protein expression (Figure 6B). Increased ERK phosphorylation was accompanied by increased total protein levels (Figure 6C). miRNA-145 overexpression did not lead to changes in Akt phosphorylation or expression (Figure 6D) whereas IκB levels increased (Figure 6E), indicating reduced NF-κB pathway signalling. Finally, p21 protein expression was increased in response to miRNA-145 overexpression (Figure 6F).

### 3.7. Bystander Effect of Conditioned Medium from miRNA-145 Overexpressing Cells

**Figure 7:**
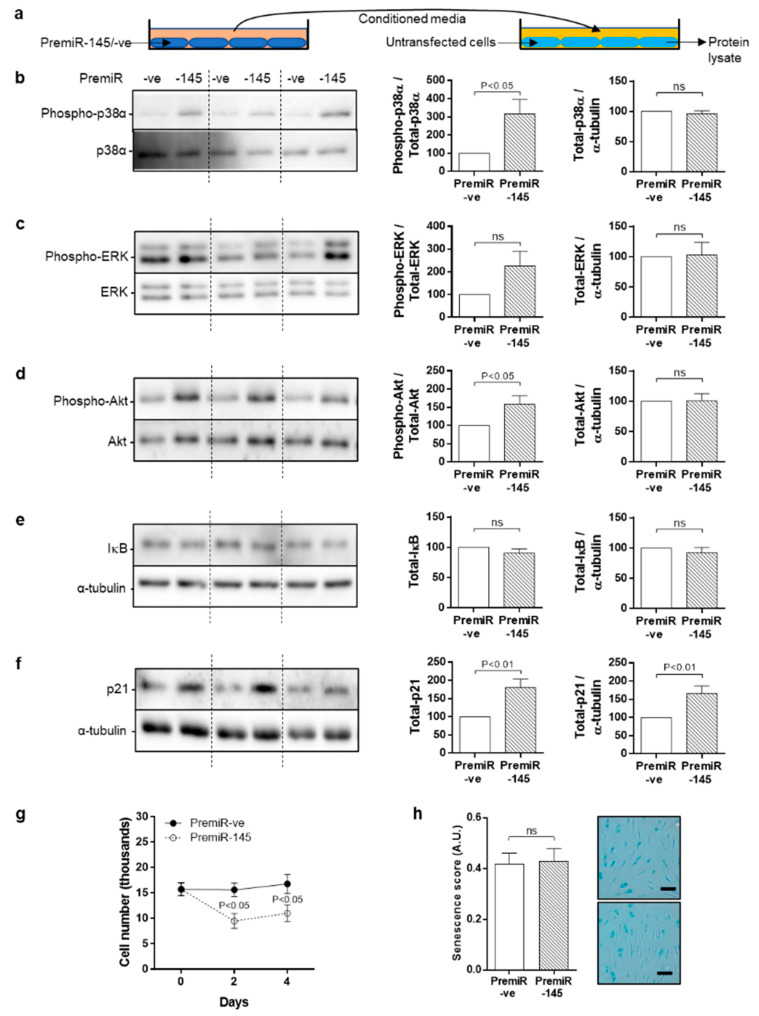
Conditioned medium from miRNA-145 overexpressing SMC induces a bystander effect in naive cells. (**a**) Cells were serum-starved for 72 h prior to exposure to conditioned (0.4% FCS) medium harvested from cells transfected with premiRNA-145 or premiRNA -ve for 96 h. Protein lysates were immunoblotted for (**b**) p38, (**c**) ERK, (**d**) Akt (all expression and phosphorylation), (**e**) IκB, (**f**) p21 expression. Phosphorylation of proteins was measured against expression of the same protein, whereas changes in expression only were measured against α-tubulin as a loading control (*n* = 6–9). (**g**) Cells were serum starved for 72hrs prior to exposure to conditioned (0.4% FCS) medium harvested from cells transfected with premiRNA-145 or premiRNA -ve for 96 h. Cell counts were performed at 48 and 96h post exposure to determine cell number or (**h**) were fixed and stained for presence of SA-β-galactosidase staining (scale bar = 100 μm; *n* = 6–9).

Senescent cells can induce senescence and DDR in neighbouring cells through paracrine mechanisms known as a “bystander effect”. ND-SMC were transfected with premiRNA –ve (control) or premiRNA-145 (overexpression) for 96 h, after which conditioned medium (CM) was collected and applied to previously serum-starved (72 h), naïve ND-SMC. After 96 h, lysates were prepared from the CM-stimulated cells and immunoblotted (Figure 7A). Again, increased phosphorylation of p38 was observed without increased total protein (Figure 7B). ERK phosphorylation was variable and inconsistent (Figure 7C). In contrast to the original miRNA-145 overexpressing cells, an increase in Akt phosphorylation was observed in the CM-treated cells in the absence of changes in total Akt (Figure 7D). IκB was unaffected (Figure 7E) and p21 protein was increased in the CM-stimulated cells, analogous to miRNA-145 transfected cells (Figure 7F).

Proliferation assays were performed over 4 days by exposing serum-starved naïve cells to CM from premiRNA –ve or premiRNA-145 transfected cells. Whilst control cells remained viable, they did not proliferate, however significant cell loss was observed in SMC exposed to CM collected from miRNA-145 overexpressing cells (Figure 7G). Both populations exhibited substantial, yet comparable degrees of senescence (Figure 7H).

## 4. Discussion

We previously discovered that saphenous vein SMC (SV-SMC) cultured from patients with T2DM displayed a persistent enlarged, flattened morphology with impaired proliferation [6] and that this was driven by an epigenetic mechanism involving elevated expression of miRNA-145 [8]. Epigenetic changes are not uniquely attributable to microRNAs but are regulated by other mechanisms, for example histone modifications and altered DNA methylation/acetylation. We propose that the acquired phenotype in human T2DM -SMC is very likely to also involve some of these epigenetic modifications, as has been reported recently in aneurysm disease [21]. We have previously explored DNA modifications in the miR-145 promoter although the data were inconclusive; further research is required using cells from larger number of patients [22]. Notwithstanding, we demonstrated conclusively that miRNA-145 is a critical regulator of phenotypic switching in human SV-SMC in T2DM [8]. MiRNA-145 is endorsed as a master regulator of SMC behaviour [23] and importantly has been identified as a tumour suppressor miRNA, confirming an association with DNA damage in a variety of cancer cell-types [24,25].

The average age of the ND and T2DM patient cohorts in the current study was in agreement with our previous report [8]; it was therefore unsurprising to reveal evidence of cellular aging in both patient populations. However, T2DM-SMC cells were unequivocally distinct from ND cells, lending weight to the idea that additional mechanisms conferring accelerated aging and senescence in T2DM may be responsible. In support of this hypothesis, we recently demonstrated in cultured human SMC of both aortic and venous origin that miRNA-145 expression levels did not correlate with chronological age but were elevated in abdominal aortic aneurysm SMC; a disorder associated with accelerated vascular aging [18].

We proceeded to explore components of the DDR and confirmed that T2DM-SMC exhibited persistent senescence and DNA damage evidenced by aberrant cell nuclei, increased phosphorylation of H2AX and increased gene expression of ATM, ATR and p21. Whilst higher mRNA expression levels of IL-8 suggested a developing SASP, this was not corroborated by IL-6 or MCP-1 mRNA expression, or increased protein secretion suggesting that T2DM-SMC had become senescent without progressing to the secretory phenotype [26,27]. It was therefore logical to explore whether inflicting DNA damage per se in ND-SMC could drive the acquisition of a native T2DM-SMC phenotype. In doing so, we confirmed comparable changes in proliferation, senescence, apical kinase and miRNA-145 gene expression. Taken together, our data indicate that T2DM-SMC exhibit enhanced DNA damage and senescence and that stimulating DNA damage in ND-SMC creates these features that are characteristic of T2DM-SMC. Our findings concur with a very recent study in which DNA damage and senescence in venous SMC was higher in T2DM versus ND patients who underwent lower limb amputation [28]. The authors revealed that the observed enhanced DNA damage in the vasculature of T2DM patients plays an important role in venous SMC calcification.

The primary inducers of the DDR signalling cascade are the ATR and ATM kinases, which are generally activated in response to single strand and double strand DNA breaks, respectively (reviewed in [29]). A third member of the family, DNA-PK, is a key component of the non-homologous end joining process that repairs double strand DNA breaks [29]. Whilst we discovered that ATM and ATR (bot not DNA-PK) were significantly elevated in T2DM-SMC, pharmacological and siRNA-mediated modulation revealed that it was only inhibition of ATR (single strand DNA breaks) that led to reduced SV-SMC proliferation. This unanticipated reduction in cell number is not without precedent and has previously been documented in a variety of cancer cell lines; reportedly by inducing apoptosis [30]. The reduction in cell number that we observed in proliferation assays may be reflective of enhanced apoptosis rather than impaired proliferation, although this was not evaluated. Nevertheless, neither pharmacological nor gene-silencing approaches to apical kinase inhibition modulated miRNA-145 expression, suggesting that the aberrant miRNA-145 expression was not directly driven by apical kinase activity and its effect on reducing cell number is mediated via an alternate mechanism. Factors within the diabetic milieu (and molecular changes within the diabetic SMC itself) are complex and involve crosstalk between multiple pathways that include but are not limited to, apical kinase expression/activity. Whilst DNA damage itself can induce miR-145 (Figure 7), we also know that other pathways, for example we previously revealed TGF-β signalling [8], which could theoretically have a greater impact on proliferation than apical kinase inhibition.

The current study has emphasized the limitations of relying on gene expression studies only. Ideally, protein levels should be evaluated although in our hands, this was not possible due to unreliable detection of the high molecular weight apical kinases. We propose that the future development of kinase activity assays would be both reliable and quantifiable.

Whilst accumulating evidence supports the idea that miRNAs may be novel players in the DDR [9], a functional link between miRNA-145, SMC senescence and T2DM has not been established until now. Overexpression of miRNA-145 increased β-galactosidase to levels comparable with those in native T2DM cells whilst expression patterns of IL-1α were not altered and LMNB1 was significantly increased, contrary to the reduction observed in native T2DM cells. However, published reports are at variance, for example knockdown of LMNB1 in mouse embryonic fibroblasts reduced miRNA-145 levels [31], whilst overexpression of miRNA-145 led to a reduction in LMNB1 in mesothelioma cells [32] but not in rat cardiac myocytes [33]. Thus, the increased LMNB1 expression that we observed in miRNA-145 overexpressing SV-SMC may not be involved in induction of senescence.

With respect to evidence for SASP, miRNA-145 overexpressing cells secreted significantly higher levels of IL-6, IL-8 and MCP-1, supporting the idea that miRNA-145 may propagate senescence in SV-SMC via autocrine or paracrine mechanisms. In contrast, a role for miRNA-145 in the initiation of DNA damage was not confirmed in terms of changes in γH2AX, ATM and p21, although elevation of ATR mRNA was significant, akin to native T2DM-SMC. Taken together, these data suggest that miRNA-145 lies downstream of the initiation of DNA damage and plays a key role in provoking/reinforcing senescence.

Whilst the DDR pathway is rapid, development of SASP is a delayed and often prolonged event over days or weeks [34,35], inferring that canonical DDR signalling is insufficient to drive SASP and that additional molecular mechanisms are necessary. Notably, the p38 MAPK pathway has been shown to be a signalling target of a variety of cellular stresses–oxidative, metabolic, DNA damage and mechanical damage [34]. Importantly, p38 sustains SASP in cancer cells and tumorigenesis [36] and has been described as a marker of vascular SMC senescence [27].

Our studies revealed chronic activation of p38α in miRNA-145 overexpressing cells. A complex, bi-directional relationship between miRNA-145 and p38 appears to exist. For example, p38 phosphorylation was suppressed by miRNA-145 in placental tissue [37], yet in a different study an increase in p38 phosphorylation was noted on overexpression and knockdown of miRNA-145 in chondrocytes [38]. A p38 response element has been identified within the promoter of miRNA-145 [39,40], and p38 increased the processing of pri-miRNA-145 to mature miRNA-145 [41].

We also observed increased ERK1/2 phosphorylation that was accompanied by increased ERK protein expression. The relationship between miRNA-145 and ERK1/2 in general appears to be inverse; indeed ERK1/2 inhibited miRNA-145 expression in human aortic SMC [42] and miRNA-145 inhibition reportedly enhanced ERK1/2 and Akt phosphorylation in cancer cells [43]. Conversely, we observed a positive relationship between miRNA-145 and ERK1/2 which might be indicative of a negative feedback mechanism. Similarly, the observed increase in the anti-inflammatory protein IκB may be a compensatory mechanism by the cell to ameliorate stress signalling. Taken together, these observations provide evidence of chronic stress in miRNA-145 overexpressing SV-SMC.

p21 is a key downstream effector of p53 in the DDR pathway, halting the cell cycle to allow DNA damage to be repaired [44]. We observed significantly elevated p21 expression in native T2DM cells which was mimicked by inducing DNA damage or over-expressing miRNA-145. This is in keeping with reduced proliferation in T2DM and miRNA-145 over-expressing cells, and concurs with previous literature where miRNA-145 and p53 have a complementary positive feedback relationship that increases p21 expression [45].

Factors released from senescent cells can inflict an autocrine or paracrine response in neighbouring cells, known as a bystander effect [46], that can further reinforce DNA damage and senescence. We showed that activation of p38 and expression of p21 were analogous to that observed in “donor” miRNA-145 overexpressing cells, suggesting that these signalling events are important for both induction and propagation of DNA damage and cellular senescence.

## 5. Conclusions

**Figure 8:**
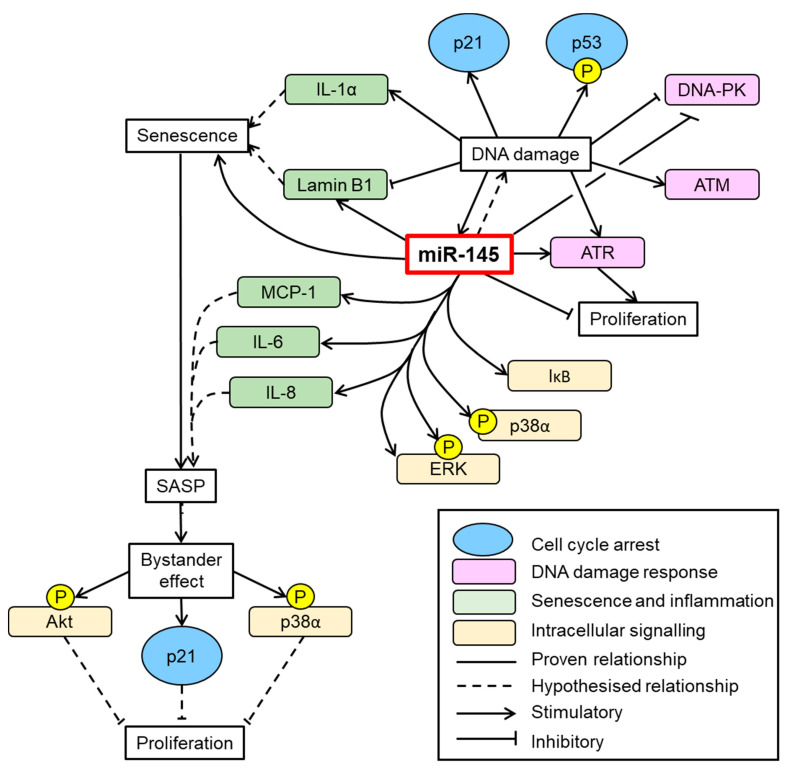
Summary of the central role for miRNA-145 in influencing SV-SMC DNA damage, proliferation and senescence. DNA damage upregulates expression of miRNA-145 in addition to inducing cell cycle arrest (p21 and p53 phosphorylation), upregulating apical kinase expression (ATR, ATM, DNA-PK) and cellular senescence (IL-1α, Lamin B1 and SA β-gal. miRNA-145 reinforces DDR by also upregulating ATR expression, triggering intracellular kinase cascades (IκB, p38α, ERK) and inducing components of SASP (MCP-1, IL-6, IL-8). This induction of senescence and SASP inflicts a paracrine bystander effect on neighbouring cells through chronic activation of Akt and p38α, which can block cellular proliferation and perpetuate the T2DM-SMC phenotype.

In summary, emerging evidence supports a role for epigenetics in VSMC senescence and aging and in particular, proposed functions for miRNAs [reviewed in [21]]. Induction and progression of vascular pathophysiological states is multifactorial. Whilst SMC plasticity is central to both adaptive (physiological) and adverse (pathological) remodelling [47], aging and senescence are singularly detrimental. Importantly, we have identified a novel mechanism linking aberrant miRNA-145 expression to SV-SMC senescence in the setting of macrovascular complications of T2DM. Figure 8 provides an illustrative summary of our findings. Succinctly, miRNA-145 drives increased senescence, reduced cell proliferation and activation of chronic stress signalling in response to DNA damage although its potential to instigate DNA damage may be secondary to these important aspects. A next logical step would be to explore whether gene silencing in native T2DM-SMC can ameliorate miR-145-driven adverse cellular mechanisms that might delay vascular decline in the setting of vein graft failure.

Whilst the use of 2D cell cultures is ideal for exploring mechanistic aspects, one of the limitations is that other contributing factors that are active in intact tissues and organisms are not considered. Our laboratory has a track record of expertise in the use of saphenous vein organ cultures where the effect of other cell types (particularly the endothelium) and extracellular matrix components in situ can also be considered [48]. Future studies will explore such interactions and their functional outcomes.

## Data Availability

Data are contained within the article and/or supplementary materials.

## Supplementary Materials

The following are available online at https://www.mdpi.com/article/10.3390/cells10040919/s1, Figure S1: Secretion of senescence-associated inflammatory cytokines. Figure S2: Stimulation of miR-145 by oxidative stress. Figure S3: siRNA knockdown of ATM, ATR and DNA-PK has a prolonged inhibition on mRNA levels. Figure S4: PremiR-145 induces expression of miR-145.
