# Supplementary material for: Role of MicroRNA-145 in DNA Damage Signalling and Senescence in Vascular Smooth Muscle Cells of Type 2 Diabetic Patients
Source: Cells. 2021 Apr 16;10(4):919. doi: 10.3390/cells10040919 (PMC8073820; doi:10.3390/cells10040919)
Supplement: Supplementary file 1 [file cells-10-00919-s001.zip › cells-1152177-supplementary.pdf]

FIGURE 1

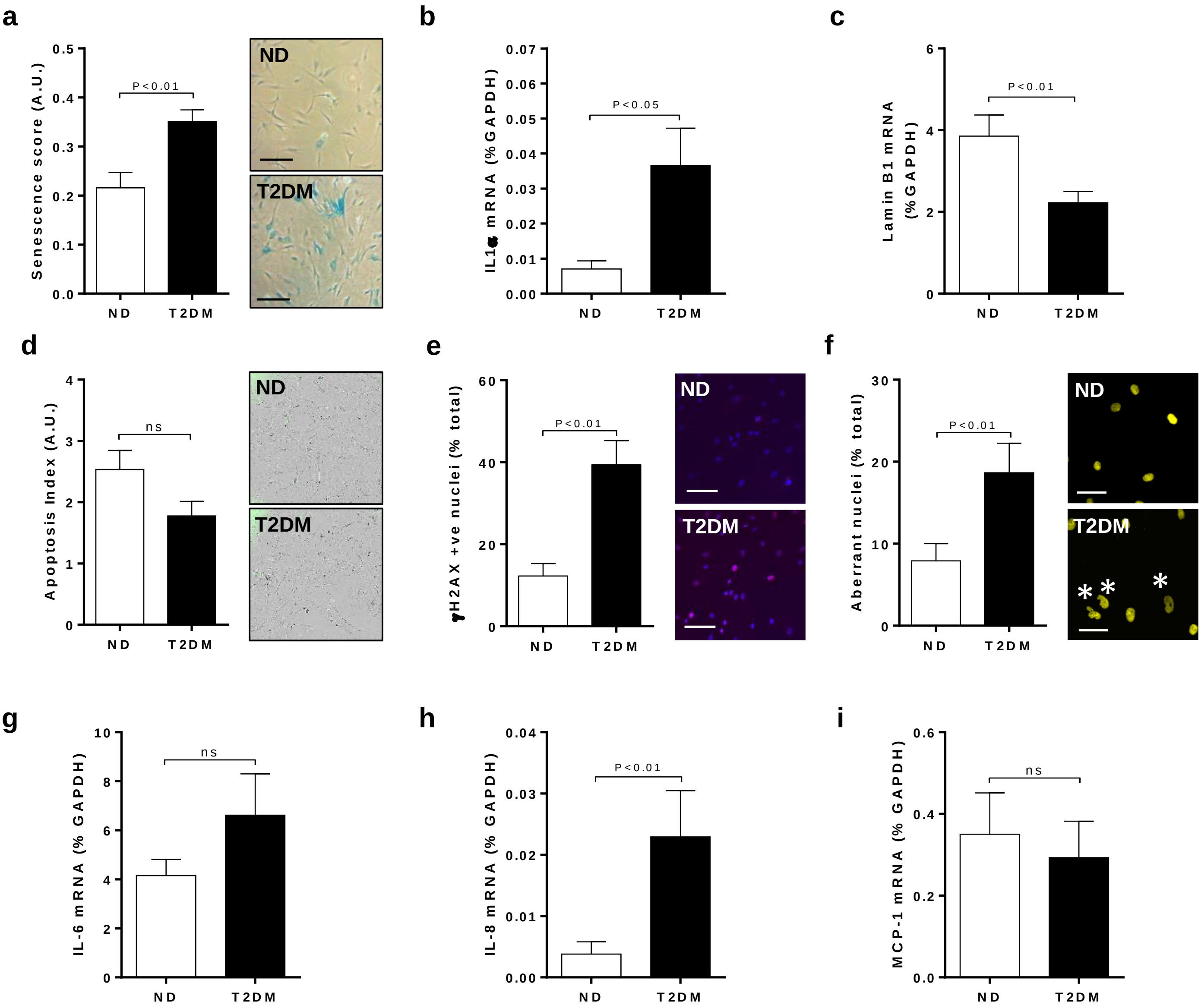

a
Senescence score (A.U.)
P<0.01
ND
T2DM
b
IL1α mRNA (%GAPDH)
P<0.05
c
Lamin B1 mRNA (%GAPDH)
P<0.01
d
Apoptosis Index (A.U.)
ns
e
γH2AX +ve nuclei (% total)
P<0.01
f
Aberrant nuclei (% total)
P<0.01
g
IL-6 mRNA (% GAPDH)
ns
h
IL-8 mRNA (% GAPDH)
P<0.01
i
MCP-1 mRNA (% GAPDH)
ns

SUPPLEMENTARY FIGURE 1

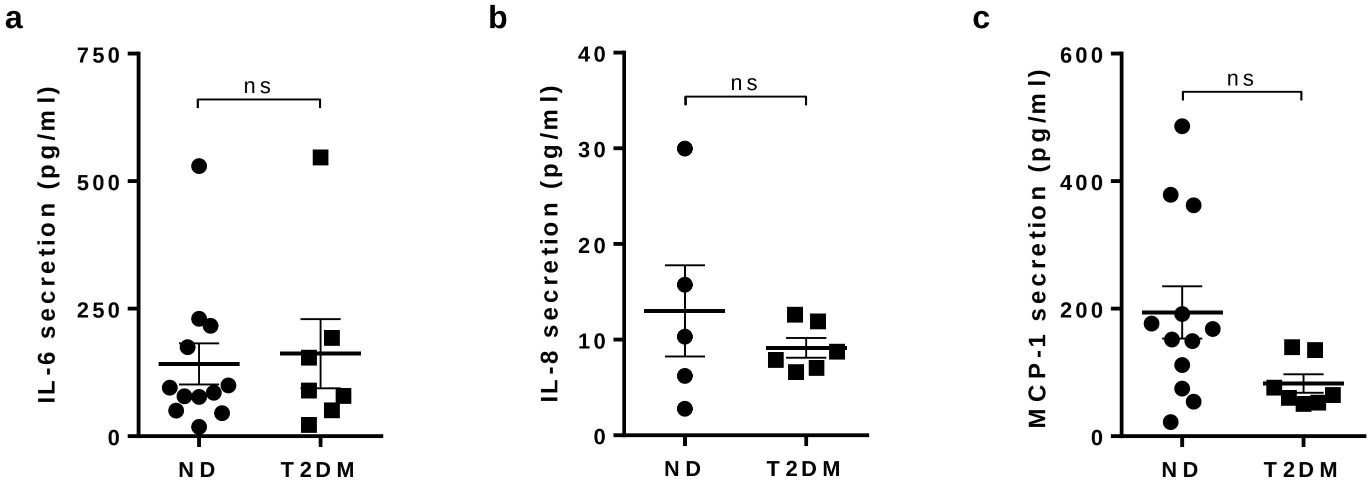

Supplementary Figure 1: Secretion of senescence-associated inflammatory cytokines. Cells were cultured in serum free media for 72 h, and the basal secretion of senescence-associated inflammatory cytokines quantified. (A) IL-6, (B) IL-8 and (C) MCP-1 were measured using ELISA (all n=5-12).

FIGURE 2

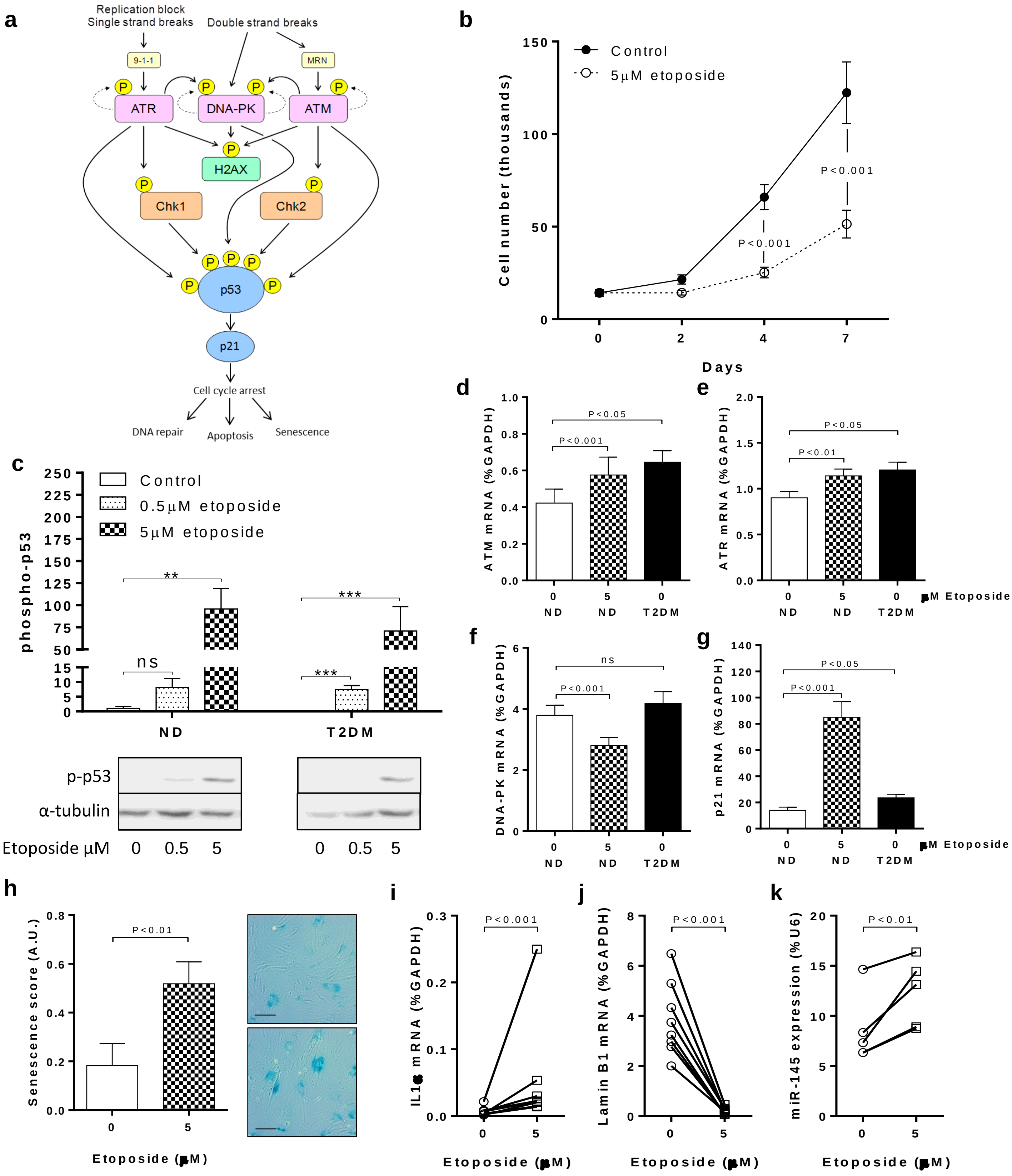

a
Replication block
Single strand breaks
Double strand breaks
9-1-1
MRN
ATR
DNA-PK
ATM
H2AX
Chk1
Chk2
p53
p21
Cell cycle arrest
DNA repair
Apoptosis
Senescence
b
Control
5μM etoposide
Cell number (thousands)
P<0.001
P<0.001
Days
c
Control
0.5μM etoposide
5μM etoposide
phospho-p53
ns
ND
T2DM
p-p53
α-tubulin
Etoposide μM
d
ATM mRNA (%GAPDH)
P<0.05
P<0.001
ND
T2DM
e
ATR mRNA (%GAPDH)
P<0.05
P<0.01
μM Etoposide
f
DNA-PK mRNA (%GAPDH)
ns
P<0.001
g
p21 mRNA (%GAPDH)
P<0.05
P<0.001
h
Senescence score (A.U.)
P<0.01
Etoposide (μM)
i
IL1β mRNA (%GAPDH)
P<0.001
j
Lamin B1 mRNA (%GAPDH)
P<0.001
k
miR-145 expression (%U6)
P<0.01

SUPPLEMENTARY FIGURE 2

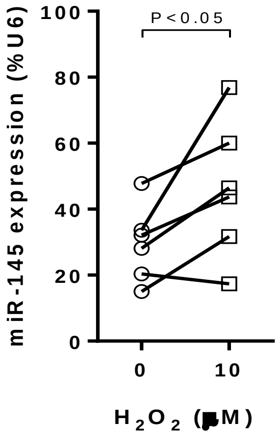

Supplementary Figure 2: Stimulation of miR-145 by oxidative stress. Cells were cultured in serum free media supplemented with 10 μM H_2O_2 for 6 h, and the expression of miR-145 quantified using RT-PCR (n=5).

FIGURE 3

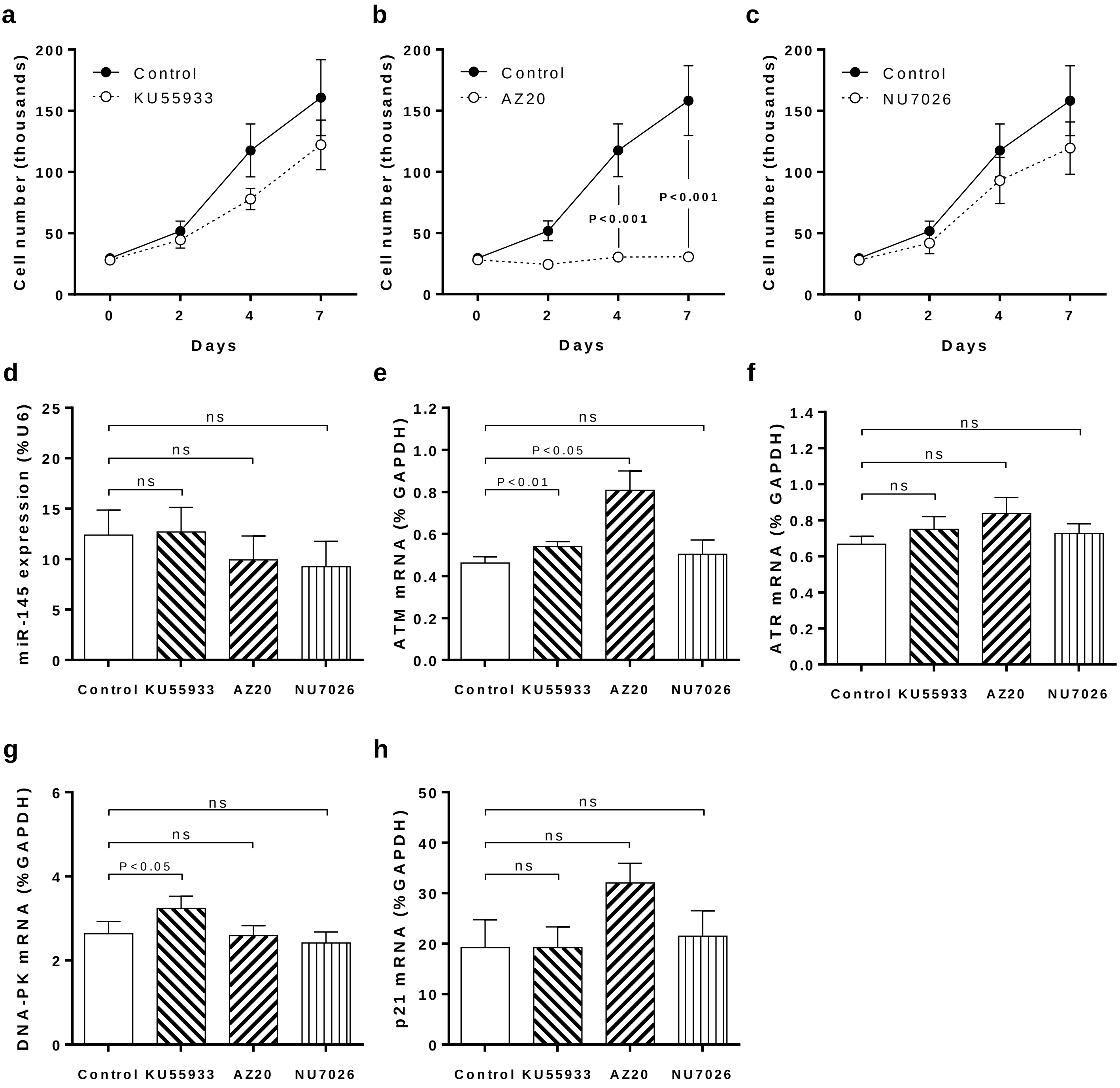
a
b
c
Control
KU55933
AZ20
NU7026
P<0.001
P<0.001
Cell number (thousands)
Days
d
e
f
g
h
miR-145 expression (%U6)
ATM mRNA (% GAPDH)
ATR mRNA (% GAPDH)
DNA-PK mRNA (%GAPDH)
p21 mRNA (%GAPDH)
ns
P<0.01
P<0.05
Control KU55933 AZ20 NU7026

SUPPLEMENTARY FIGURE 3

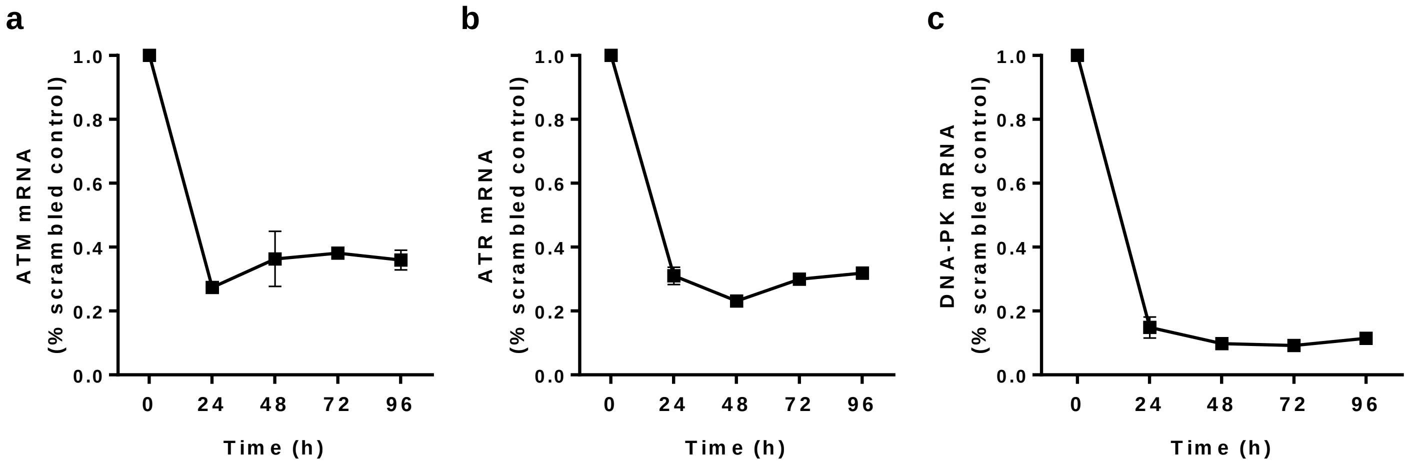

Supplementary Figure 3: siRNA knockdown of ATM, ATR and DNA-PK has a prolonged inhibition on mRNA levels. Cells were transfected with 20nM siRNA for 6 hours. RNA was harvested at 24, 48, 72 and 96 h to confirm levels of knockdown for (A) ATM, (B) ATR and (C) DNA-PK (all n=2).

FIGURE 4

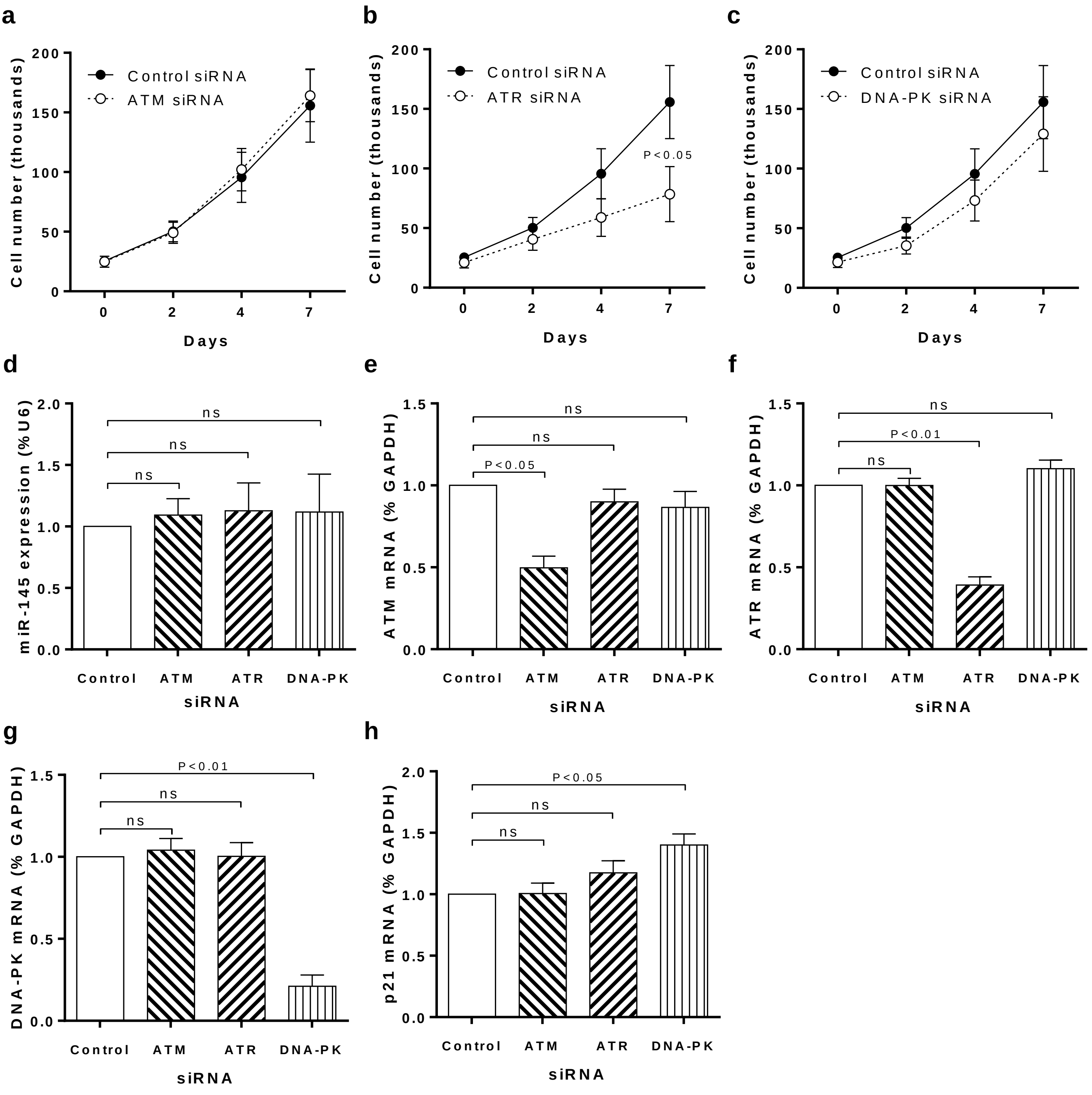

a
Control siRNA
ATM siRNA
Cell number (thousands)
Days
b
Control siRNA
ATR siRNA
P<0.05
c
Control siRNA
DNA-PK siRNA
d
miR-145 expression (%U6)
ns
siRNA
Control
ATM
ATR
DNA-PK
e
ATM mRNA (% GAPDH)
P<0.05
f
ATR mRNA (% GAPDH)
P<0.01
g
DNA-PK mRNA (% GAPDH)
P<0.01
h
p21 mRNA (% GAPDH)
P<0.05

SUPPLEMENTARY FIGURE 4

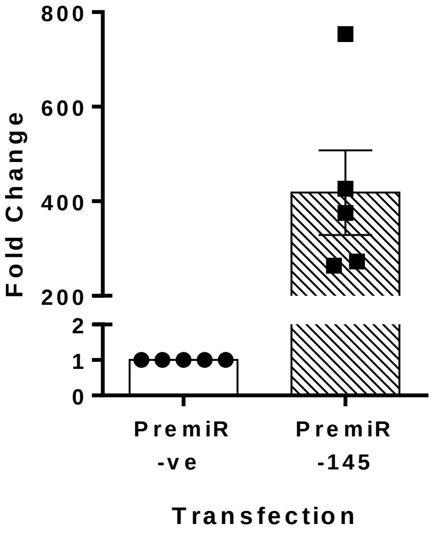

Supplementary Figure 4: PremiR-145 induces expression of miR-145. Cells were transfected with 15 nM siRNA for 6 hours. RNA was harvested at 48 h to confirm induction of miR-145 (n=5).

FIGURE 5

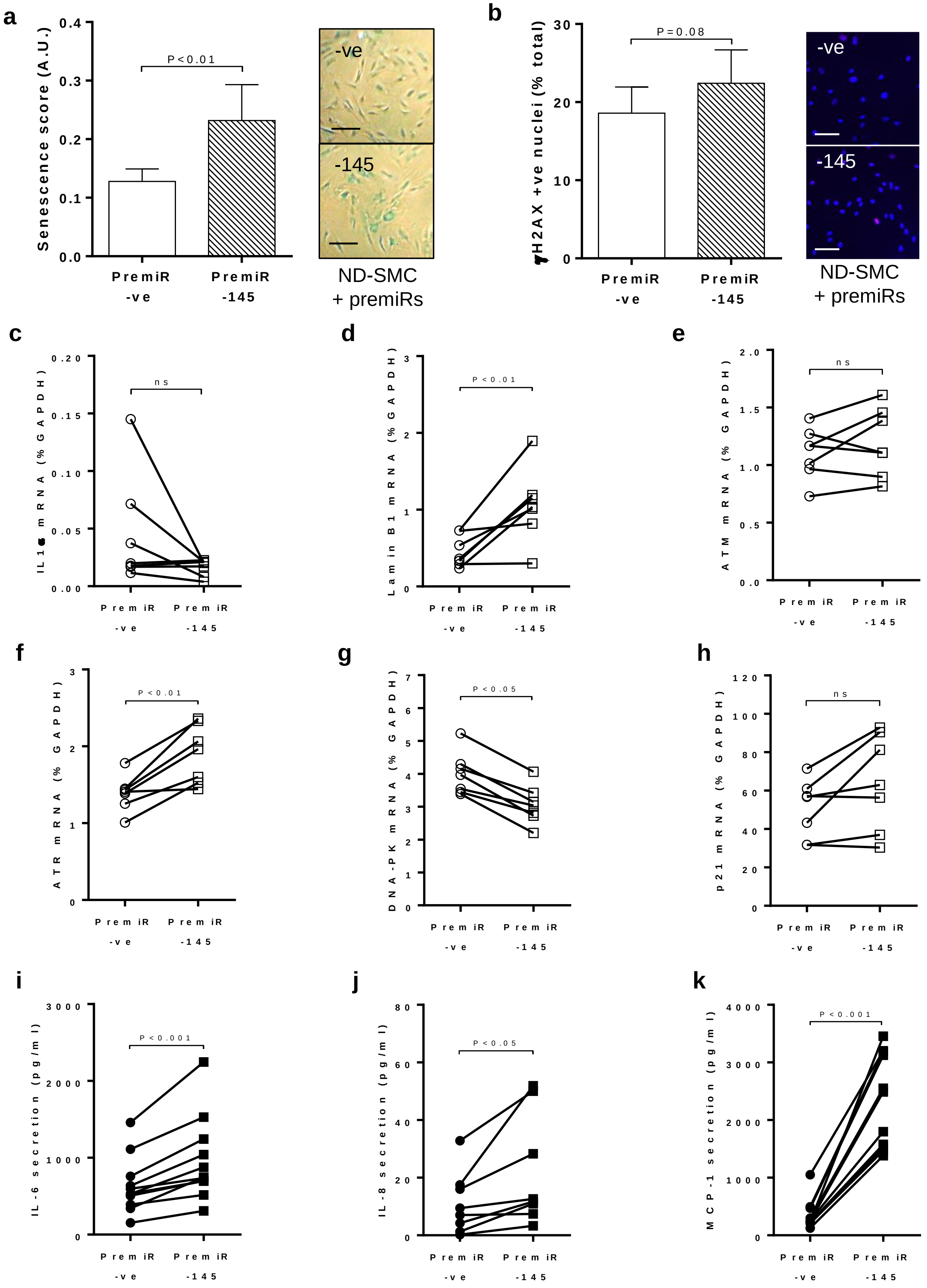
a
Senescence score (A.U.)
P<0.01
PremiR -ve
PremiR -145
-ve
-145
ND-SMC + premiRs
b
γH2AX +ve nuclei (% total)
P=0.08
PremiR -ve
PremiR -145
ND-SMC + premiRs
c
IL1α mRNA (% GAPDH)
ns
d
Lamin B1 mRNA (% GAPDH)
P<0.01
e
ATM mRNA (% GAPDH)
ns
f
ATR mRNA (% GAPDH)
P<0.01
g
DNA-PK mRNA (% GAPDH)
P<0.05
h
p21 mRNA (% GAPDH)
ns
i
IL-6 secretion (pg/ml)
P<0.001
j
IL-8 secretion (pg/ml)
P<0.05
k
MCP-1 secretion (pg/ml)
P<0.001

FIGURE 6

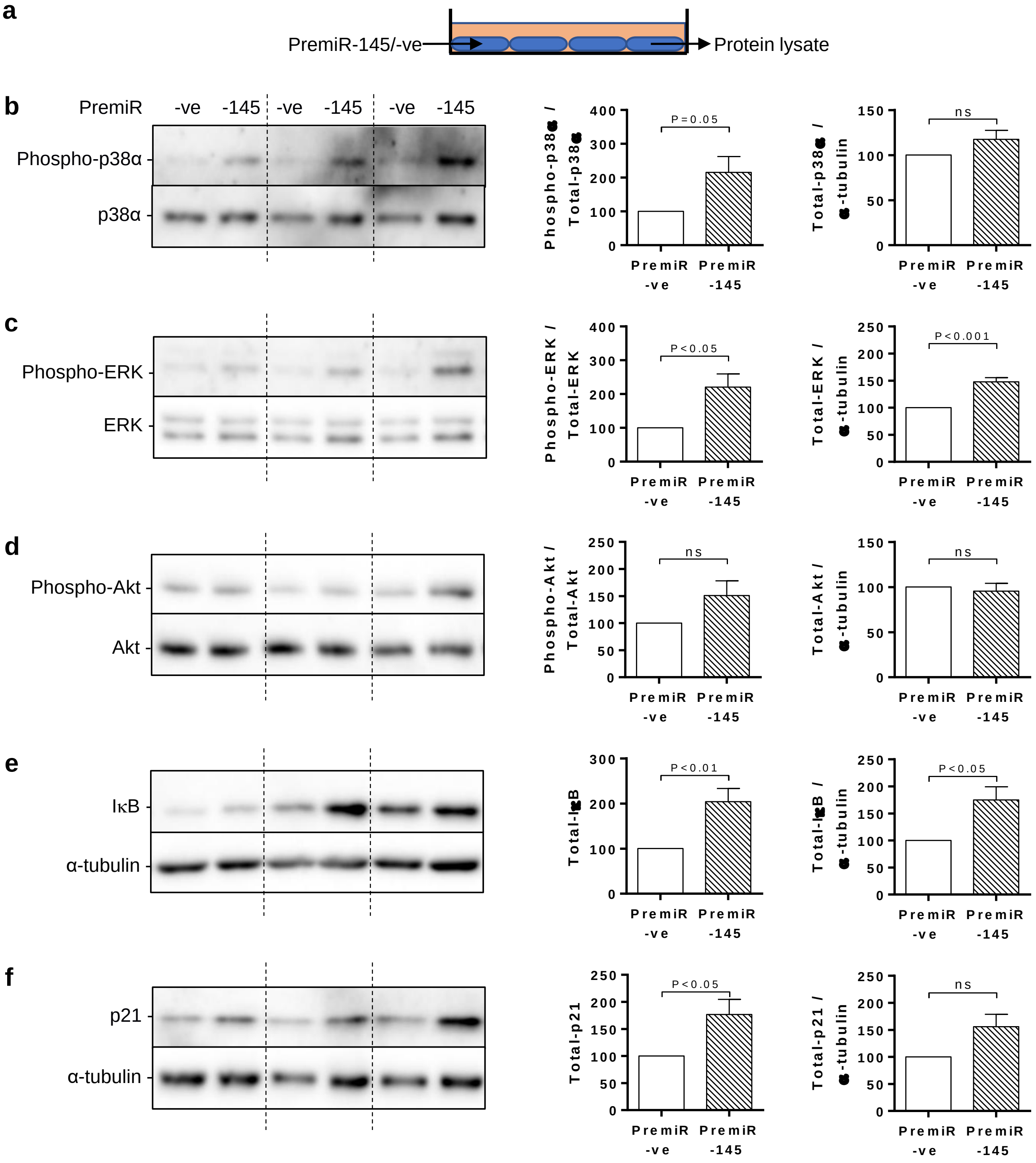
a
PremiR-145/-ve
Protein lysate
b
PremiR
-ve
-145
Phospho-p38α
p38α
c
Phospho-ERK
ERK
d
Phospho-Akt
Akt
e
IκB
α-tubulin
f
p21
α-tubulin
P=0.05
P<0.05
P<0.001
P<0.01
ns
PremiR -ve
PremiR -145
Phospho-ERK / Total-ERK
Total-ERK / α-tubulin
Phospho-Akt / Total-Akt
Total-Akt / α-tubulin
Total-p21
Total-p21 / α-tubulin

FIGURE 7

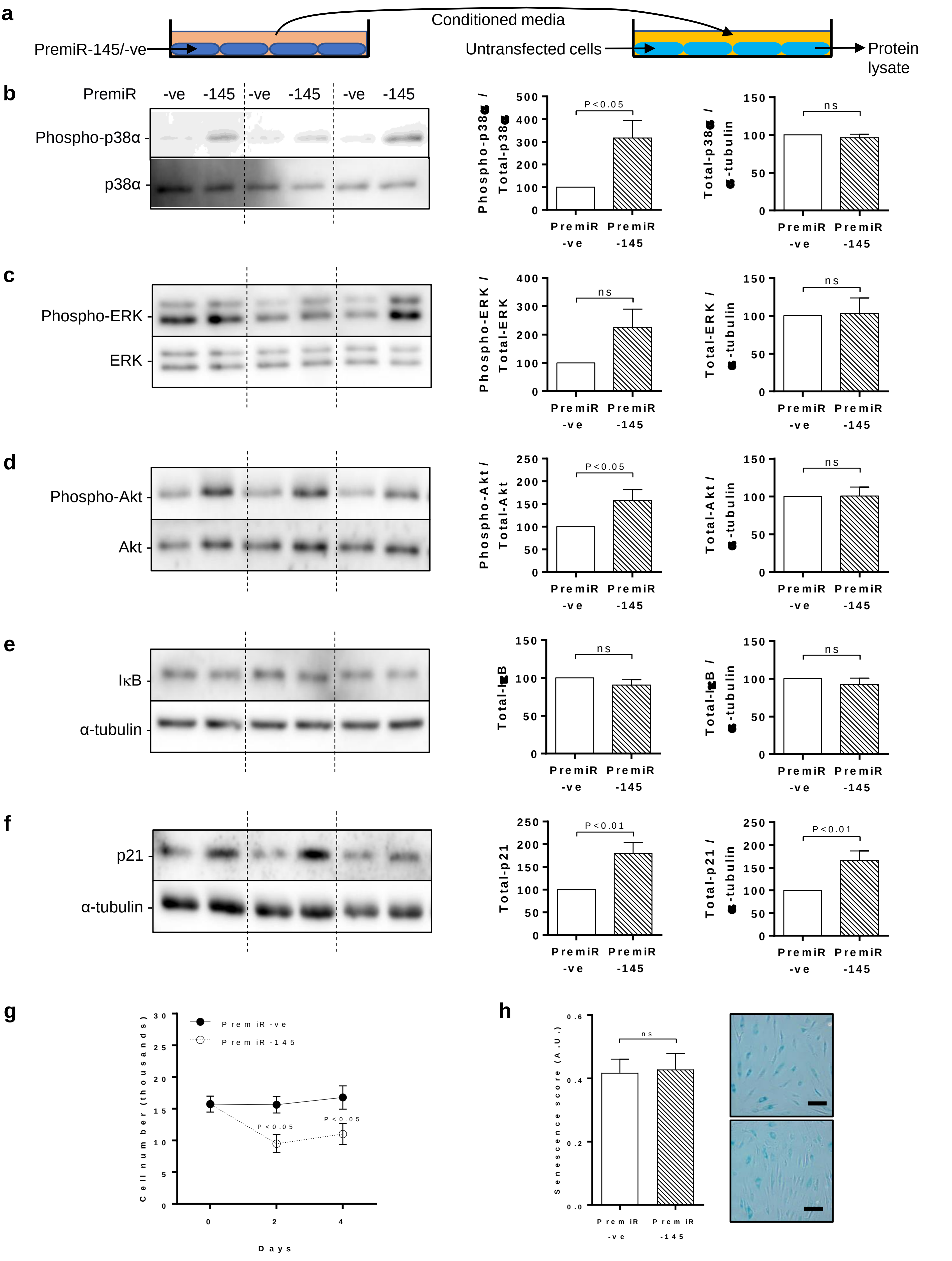
a
Conditioned media
PremiR-145/-ve
Untransfected cells
Protein lysate
b
PremiR
-ve
-145
Phospho-p38α
p38α
Phospho-p38α / Total-p38α
P<0.05
Total-p38α / α-tubulin
ns
PremiR -ve
PremiR -145
c
Phospho-ERK
ERK
Phospho-ERK / Total-ERK
Total-ERK / α-tubulin
d
Phospho-Akt
Akt
Phospho-Akt / Total-Akt
Total-Akt / α-tubulin
e
IκB
α-tubulin
Total-IκB
Total-IκB / α-tubulin
f
p21
Total-p21
Total-p21 / α-tubulin
P<0.01
g
PremiR-ve
PremiR-145
Cell number (thousands)
Days
P<0.05
h
Senescence score (A.U.)

FIGURE 8

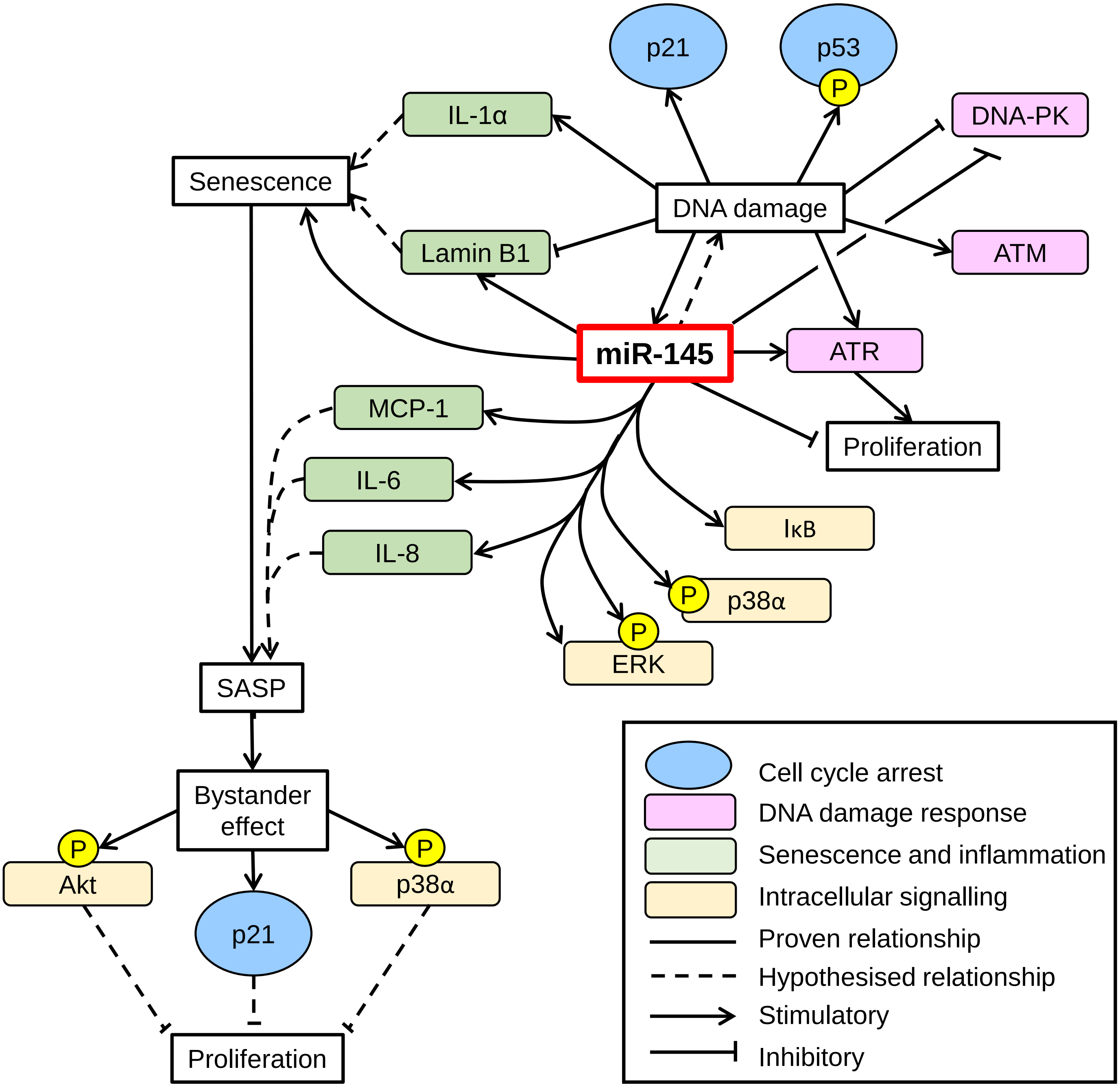
p21
p53
P
IL-1α
DNA-PK
Senescence
DNA damage
Lamin B1
ATM
miR-145
ATR
MCP-1
Proliferation
IL-6
IκB
IL-8
P
p38α
P
ERK
SASP
Bystander
effect
P
Akt
P
p38α
p21
Proliferation
Cell cycle arrest
DNA damage response
Senescence and inflammation
Intracellular signalling
Proven relationship
Hypothesised relationship
Stimulatory
Inhibitory
